# Plasma GFAP and NLRP3 Associate with Cognitive Impairment After Recent Small Subcortical Infarct via Periventricular White Matter Hyperintensity

**DOI:** 10.1002/cns.70780

**Published:** 2026-02-17

**Authors:** Tianxiang Lan, Chunhua Wang, Yuying Yan, Tang Yang, Jingyu Cui, Rumei Lei, Rongfeng Luo, Shuai Jiang, Bo Wu

**Affiliations:** ^1^ Department of Neurology, West China Hospital Sichuan University Chengdu China; ^2^ Department of Radiology, Sichuan Clinical Research Center for Cancer, Sichuan Cancer Hospital & Institute, Sichuan Cancer Center University of Electronic Science and Technology of China Chengdu China; ^3^ Centre of Cerebrovascular Diseases, West China Hospital Sichuan University Chengdu China

**Keywords:** biomarkers, cerebral small vessel diseases, cognitive impairment, neuroimaging, plasma proteins

## Abstract

**Aim:**

To assess whether early plasma inflammatory proteins identify SVD‐related stroke patients at high risk of cognitive impairment and to examine imaging‐mediated effects.

**Methods:**

This single‐center retrospective study (April 2020–August 2024) enrolled patients with MRI‐confirmed recent small subcortical infarct (RSSI) and healthy controls. GFAP and NLRP3 levels were measured by ELISA. MRI markers of small vessel disease—including white matter hyperintensities (WMH), lacunes, perivascular spaces, and microbleeds—were evaluated. Cognitive function was assessed using the Montreal Cognitive Assessment (MoCA) and functional outcome by the modified Rankin Scale (mRS) at 3 months. Associations were analyzed using correlation, regression, and mediation analyses.

**Results:**

A total of 108 RSSI patients and 47 controls were included (mean age 57.9 ± 10.5 years; 64.1% male). RSSI patients had significantly higher plasma GFAP and NLRP3 levels (*p* < 0.05). Both proteins were inversely associated with total MoCA scores and cognitive subdomains, but not with 3‐month mRS. GFAP and NLRP3 correlated positively with periventricular WMH (PWMH). Mediation analysis showed that PWMH accounted for 23.88%–28.10% of the association between plasma protein levels and cognitive impairment.

**Conclusions:**

Elevated plasma GFAP and NLRP3 are associated with post‐stroke cognitive impairment in RSSI, partially mediated by PWMH. These biomarkers may help identify patients at risk of early cognitive decline.

## Graphical abstract

Ischemic stroke, recent small subcortical infarcts, induce systemic inflammation, leading to elevated levels of circulating inflammatory proteins. Through blood–brain barrier disruption, these proteins may gain access to the central nervous system and amplify microvascular injury and white matter degeneration, which directly and indirectly lead to cognition impairment.

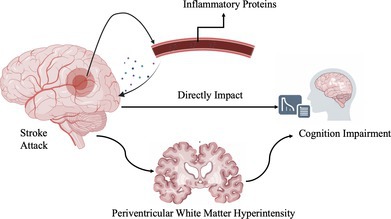

## 1 Introduction

Cognitive impairment is a complex and multifactorial disorder. Among the most extensively investigated causes are Alzheimer's disease (AD) and vascular cognitive impairment (VCI). VCI is often associated with cerebral small vessel disease (SVD), and previous studies have established that early neuroimaging features of SVD—such as white matter hyperintensities and brain atrophy—are closely linked to cognitive decline [1]. However, whether specific circulating proteins are associated with small vessel stroke or VCI remains undetermined.

Recent small subcortical infarct (RSSI), a subtype of SVD, are primarily caused by the occlusion of small penetrating arterioles [2]. Their small caliber and deep location make rapid reperfusion via mechanical thrombectomy challenging. Although individual infarcts may be small and clinical symptoms mild or atypical, cumulative damage can lead to significant neurologic deficit. A recent study has found that RSSI may induce cognitive impairment [3] [4].

Post‐stroke brain injury also provokes local and systemic immune and inflammatory responses. Ischemic damage induces the release of damage‐associated molecular patterns, which activate local immune cells such as microglia and astrocytes [5]. Concurrently, increased blood–brain barrier (BBB) permeability allows inflammatory molecules to enter the systemic circulation, recruiting peripheral immune cells and initiating complex systemic immune responses [6] [7]. Previous studies have shown that heightened post‐stroke inflammatory responses and elevated biomarker levels are closely associated with poor clinical outcomes [8].

Among candidate inflammatory markers, glial fibrillary acidic protein (GFAP)—an intermediate filament protein of astrocytes—not only maintains cellular structure but also participates in BBB repair and glial scar formation after brain injury. Elevated GFAP levels, indicative of astrocyte activation, have been shown to correlate positively with baseline National Institutes of Health Stroke Scale (NIHSS) scores, suggesting an association with more severe symptoms and a worse prognosis [9]. The NOD‐, LRR‐ and pyrin domain‐containing protein 3 (NLRP3) inflammasome is another inflammatory protein, predominantly expressed in astrocytes and microglia. It promotes the maturation and release of pro‐inflammatory cytokines (e.g., IL‐1β, IL‐18) and can induce pyroptotic cell death. Early inhibition of NLRP3 expression can mitigate inflammation, stabilize the BBB, and reduce ischemia–reperfusion injury [10] [11]. The prior animal model has demonstrated that in the brain of SVD rats, the NF‐κB/NLRP3 axis prompts the downstream activation of astrocytes [12]. However, outcomes led by these elevated proteins in the SVD population have not been figured out.

Therefore, this study aimed to investigate the early changes in these inflammation‐related proteins (GFAP and NLRP3) in patients with RSSI, analyze their association with cognitive decline, and βevaluate the potential mediating role of SVD neuroimaging markers in this relationship.

## 2 Method

This is a retrospective study, and patients were drawn from the prospective RSSI cohort conducted by the Department of Neurology at West China Hospital between April 2020 and August 2024 [13]. Eligible patients met the following criteria: (1) age ≥ 18 years old; (2) MRI‐confirmed RSSI; (3) able to finish the cognitive testing; (4) availability of blood samples for protein measurement; (5) a pre‐stroke modified Rankin Scale (mRS) score of 0–2. Exclusion criteria were: (1) prior diagnosis of AD; (2) ≥ 50% ipsilateral internal carotid stenosis, non‐atherosclerotic vasculopathies (e.g., dissection, vasculitis, moyamoya disease), or cardioembolic sources (e.g., atrial fibrillation, valvular disease, endocarditis, patent foramen ovale) identified by imaging or cardiac evaluation; (3) intracranial hemorrhage; (4) unqualified sample plasma; (5) missing imaging data. Details have been described previously [14].

The study was approved by the Ethics Committee of West China Hospital (2018521) and conducted in accordance with the Declaration of Helsinki. Written informed consent was obtained from each patient.

### 2.1 Sample Collection and Biomarker Measurement

Blood samples were collected within the first 48 h after admission. Samples were centrifuged at 1800 rpm for 15 min at 26°C to separate the serum from the blood cells. The serum sample was stored in aliquoted cryotubes at −80°C until analysis.

The concentrations of plasma GFAP and NLRP3 were measured in triplicate using commercial enzyme‐linked immunosorbent assay (ELISA) kits (FineTest, Wuhan, China; Catalog Number: EH0410 for GFAP and EH4202 for NLRP3), following the manufacturer's instructions. The protein concentrations in each plate were calculated based on the standard curves and dilution factors. The lower limit detection for GFAP is 0.313 ng/mL, and for NLRP3 is 0.781 ng/mL. Both inter‐assay and intra‐assay coefficients of variation were less than 5%.

All samples were analyzed in a single batch, and only the sample identification numbers were provided to the laboratory personnel.

### 2.2 Data Collection

Demographic and clinical characteristics were collected at admission, including age, sex, smoking, drinking assumption, history of stroke or transient ischemic attack, hypertension, diabetes mellitus, cardiovascular disease, atrial fibrillation, and family history. The NIHSS score and mRS were also collected at the baseline. For patients diagnosed with RSSI, we conducted a follow‐up by telephone or face‐to‐face to collect mRS on day 90.

### 2.3 Cognitive Assessment

Cognition was assessed by the Beijing version of the Montreal Cognitive Assessment (MoCA‐BJ) after clinical stabilization of RSSI, typically within one month after stroke onset [15]. The assessment includes visuospatial and executive ability, naming, memory, attention, language, abstraction, delayed recall, and orientation. The total score and each item score were recorded.

### 2.4 Imaging Acquisition and Assessment

All patients underwent head CT upon admission to exclude intracerebral hemorrhage. Once their condition had stabilized, baseline MRI examinations were performed on a research‐dedicated 3.0 T scanner (MAGNETOM Trio, Siemens, Erlangen, Germany) equipped with a 32‐channel head coil. The standardized imaging protocol included T1‐weighted, T2‐weighted, fluid‐attenuated inversion recovery (FLAIR), diffusion‐weighted imaging (DWI), three‐dimensional time‐of‐flight magnetic resonance angiography (TOF‐MRA), and susceptibility‐weighted imaging (SWI). Detailed sequence parameters have been previously described [16] [17].

Imaging biomarkers were assessed by two trained neurologists, with discrepancies resolved by a professional radiologist. The imaging assessors were blinded to participants' baseline characteristics, cognitive performance, and plasma biomarker levels. All the SVD neuroimaging markers were defined by STRIVE2 [18]. RSSI was defined as an infarct in the territory of a perforating arteriole with clinical manifestations corresponding to the lesion area. White matter hyperintensity (WMH) is divided into deep white matter hyperintensity (DWMH) and periventricular white matter hyperintensity (PWMH), which are both hyperintensities in T2 and T2 FLAIR in white matter. DWMH and PWMH are scaled by the Fazekas score, ranging from 0 to 3, with higher scores indicating higher severity. Lacune is round or ovoid, located at a subcortical, fluid‐filled cavity, which is a low low‐intensity signal surrounded by ring‐like hyperintensity on T2 FLAIR, and the diameter is mostly less than 15 mm. Perivascular spaces (PVS) were identified as cerebrospinal fluid‐like T2 signals, generally < 3 mm; PVS burden was graded 0–4 by lesion count (0 = none; 1 = 1–10; 2 = 11–20; 3 = 21–40; 4 ≥ 40) and assessed separately in the basal ganglia and centrum semiovale. Cerebral microbleed (CMB) is generally a round lesion, 2–5 mm in diameter, with low signal on SWI. The summary SVD score (range 0–4) was derived by awarding one point for WMH if Fazekas ≥ 2 (either PWMH or DWMH), one point for basal ganglia PVS grade ≥ 2, and one point each for the presence of lacunes and CMBs; higher scores indicate greater SVD burden.

### 2.5 Statistical Analysis

For continuous variables, we summarized the mean, standard deviation (SD), minimum, maximum, median, upper quartile, and lower quartile. For categorical variables, frequency and percentage were reported. Plasma GFAP and NLRP3 concentrations were tested for normality, and non‐normally distributed variables were log‐transformed prior to analysis. Log‐transformed GFAP and NLRP3 levels were compared between patients and controls using the Mann–Whitney U test. To further evaluate their prognostic value, multivariable ordinal logistic regression models were applied to examine the associations between log‐transformed GFAP and NLRP3 and 90‐day functional outcomes, as measured by mRS. Covariates included age, sex, smoking and drinking status, history of stroke or transient ischemic attack, and the presence of hypertension, diabetes mellitus, and hyperlipidemia. All statistical analyses involving cognitive function as the variable also included the years of education as a covariate.

To identify candidate pathways for mediation, a stepwise regression strategy was applied. First, multivariable regression models were used to evaluate the associations between protein concentrations and cognitive outcomes. Second, associations between protein concentrations and SVD imaging markers were examined. Third, the relationships between imaging markers and cognitive outcomes were assessed while adjusting for protein levels. All regression models were adjusted for predefined demographic and vascular covariates.

Imaging markers that were significantly associated with both protein levels and cognitive outcomes were subsequently entered into mediation analyses. Mediation models were constructed with log‐transformed protein concentrations as independent variables, imaging markers as mediators, and cognitive scores as outcomes, while adjusting for the same set of covariates in both the mediator and outcome models. The average causal mediation effect (ACME), average direct effect (ADE), total effect, and proportion mediated were estimated using nonparametric bootstrapping with 1000 simulations.

Missing data were handled using multiple imputation via the mice package (Multivariate Imputation by Chained Equations) in R software to minimize bias and maximize statistical power. Two‐tailed *p* values < 0.05 were considered statistically significant. All statistical analyses were conducted using R software (version 4.4.2).

## 3 Result

The baseline characteristics of the study population are summarized in Table 1. A total of 108 RSSI patients and 47 healthy controls were enrolled. The mean age of participants was 57.9 ± 10.5 years, and 64.1% were male. The median onset‐to‐admission interval was 5 days (range, 3 h–28 days). The median time for MoCA assessment from stroke onset was 28 days (28–30), and the longest time is 49 days. The median MoCA score was 23. Among the MoCA subdomains, most participants scored highest in orientation and lowest in delayed recall. Regarding SVD imaging markers, and the average summary SVD score was 1 ± 1. Perivascular spaces were more frequently observed in the centrum semiovale (PVS‐CSO) than in the basal ganglia (PVS‐BG).

**TABLE 1:** Baseline characteristics.

Characteristics	
Age, Mean (SD)	57.9 (10.5)
Sex, male, *n* (%)	100 (64.1%)
Smoke status, *n* (%)	50 (32.1%)
Alcohol assumption, *n* (%)	44 (28.2%)
Hypertension, *n* (%)	70 (44.9%)
Diabetic Mellitus, *n* (%)	44 (28.2%)
Hyper‐lipidemia, *n* (%)	28 (18.0%)
History of stroke or TIA, *n* (%)	10 (6.4%)
CVD, *n* (%)	4 (2.6%)
Years of education, years, Median (IQR)	9 (6–15)
Onset‐to‐admission interval, day, Median (IQR)	5 (3 h—28 days)
NIHSS, Median (IQR)	3 (1–6)
mRS, Median (IQR)	2 (1–4)
Time from stroke onset to MoCA assessment, day, Median (IQR)	28 (28–30)
MoCA, Median (IQR), MoCA subdomains, Median (IQR)	24 (20–26)
Visuospatial and executive ability	4 (3–5)
Naming	3 (2–3)
Attention	6 (5–6)
Language	2 (1–3)
Abstraction	1 (1–2)
Delayed recall	2 (0–3)
Orientation	6 (5–6)
Imaging markers	
Lacunes, Mean (SD)	1.2 (2.6)
PWMH, Median (IQR)	1 (1–2)
DWMH, Median (IQR)	1 (1–2)
PVS‐CSO, Median (IQR)	2 (2–3)
PVS‐BG, Median (IQR)	1 (1–2)
CMBs, Mean (SD)	2.4(8.3)
Summary SVD Score, Median (IQR)	1 (0–2)

Abbreviations: CMBs, cerebral microbleeds; CVD, cardiovascular disease; DWMH, deep white matter hyperintensity; MoCA, montreal cognitive assessment; mRS, modified rankin score; PVS‐BG, perivascular space of basal ganglia; PVS‐CSO, perivascular space of centrum semiovale; PWMH, periventricular white matter hyperintensity; SVD, small vessel disease; TIA, transient ischemic attack.

**FIGURE 1:**
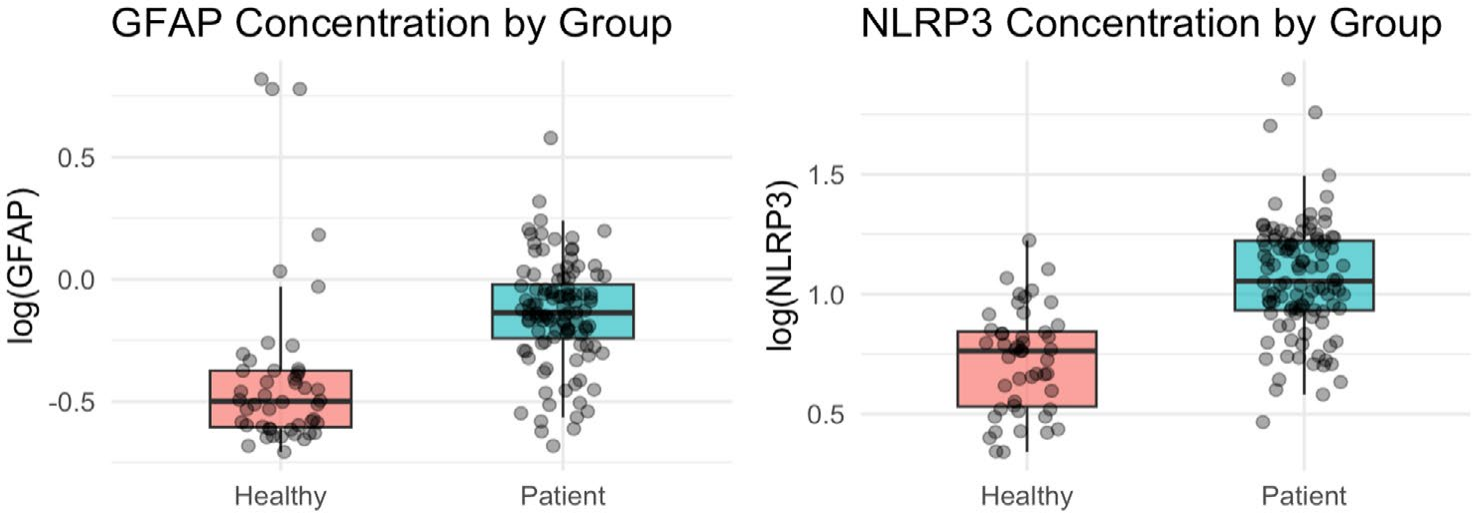
Concentration of plasma GFAP and NLRP3 after log‐transformation compared between healthy control and patients. This figure shows that compared with healthy controls, patients had significantly higher level of plasma GFAP and NLRP3.

**FIGURE 2:**
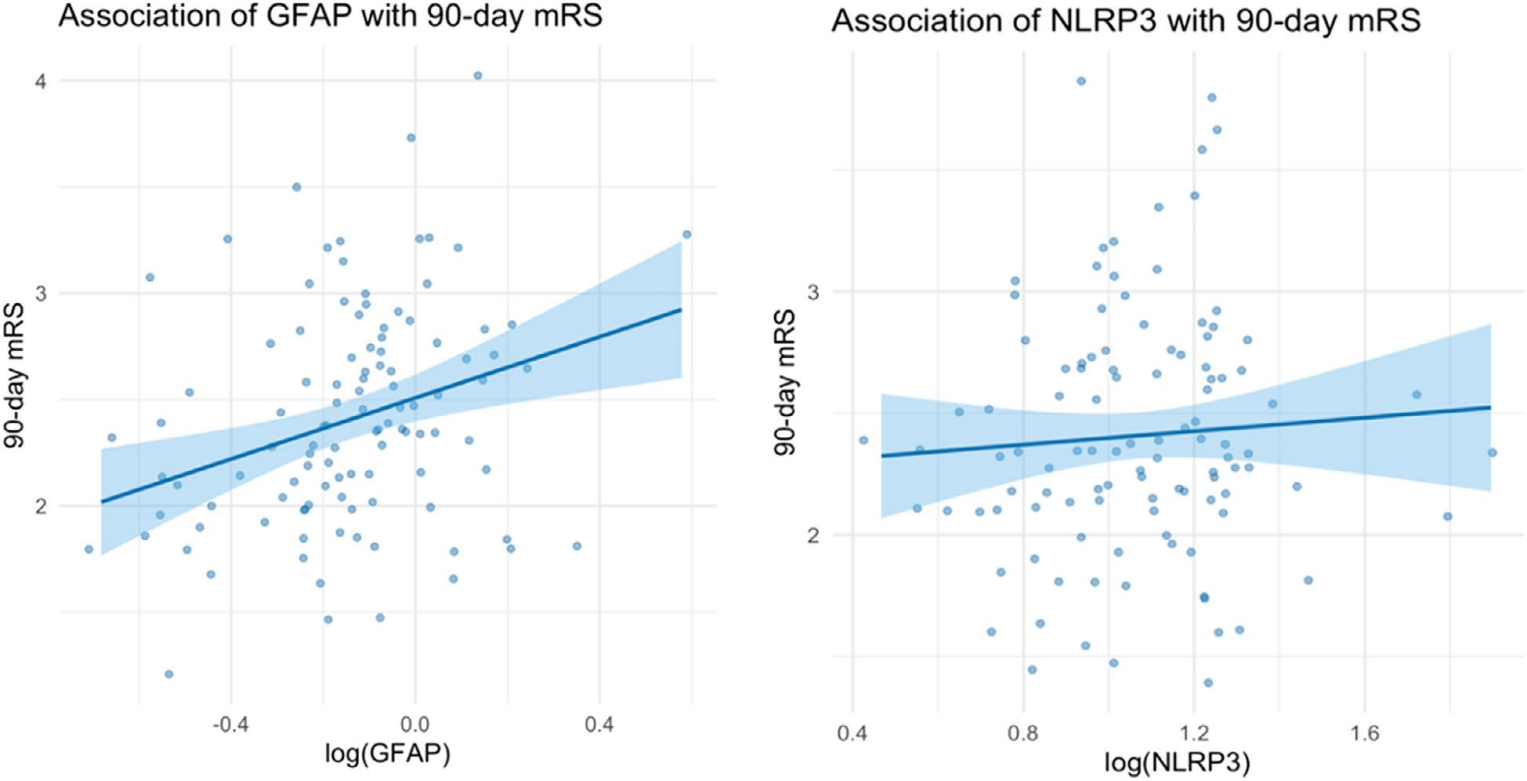
Correlation of 90‐day mRS with serum GFAP and NLRP3. The figure demonstrates that the level of plasma GFAP and NLRP3 were both not associated with 90‐day functional outcome.

Compared with healthy controls, patients with RSSI exhibited significantly elevated plasma levels of both GFAP and NLRP3. After log transformation, plasma GFAP levels were significantly higher in patients than in controls (−0.137 vs. −0.500, *p* < 0.05), corresponding to geometric mean concentrations of 0.729 and 0.316, respectively. A similar trend was observed for NLRP3 (1.054 vs. 0.764, *p* < 0.05; geometric mean concentrations corresponded to 11.324 and 5.808) (Figure 1). However, subsequent multivariable logistic regression analysis revealed that neither GFAP nor NLRP3 was independently associated with a 90‐day functional outcome as measured by the modified Rankin Scale (GFAP: OR = 4.645, 95% CI: 0.822–27.112, *p* = 0.080; NLRP3: OR = 1.676, 95% CI = 0.363–7.997, *p* = 0.512). (Figure 2).

Correlation analyses demonstrated a robust inverse association between GFAP concentrations and cognitive performance. GFAP levels were inversely correlated with the total MoCA score (β = −3.166, *p* = 0.012) and with multiple cognitive domains, including visuospatial/executive ability (β = −0.945, *p* = 0.008), naming (β = −0.646, *p* = 0.003), language (β = −0.527, *p* = 0.062), abstraction (β = −0.603, *p* = 0.002). In contrast, NLRP3 levels initially showed significant negative correlations with MoCA total score (β = −2.928, *p* = 0.034) and naming (β = −0.522, *p* = 0.032), language (β = −0.524, *p* = 0.090), abstraction (β = −0.371, *p* = 0.093) (Table 2).

**TABLE 2:** Relationship of plasma proteins and cognition.

Plasma Protein	Cognitive Domains	Beta	*p*
GFAP			
	MoCA	−3.166	0.012*
	Visuospatial and executive ability	−0.945	0.008*
	Naming	−0.646	0.003*
	Attention	−0.472	0.150
	Language	−0.527	0.062
	Abstraction	−0.603	0.002*
	Delayed recall	−0.075	0.877
	Orientation	0.288	0.692
NLRP3			
	MoCA	−2.928	0.034*
	Visuospatial and executive ability	−0.078	0.844
	Naming	−0.522	0.032*
	Attention	−0.143	0.691
	Language	−0.524	0.090
	Abstraction	−0.371	0.093
	Delayed recall	−0.417	0.435
	Orientation	−0.556	0.483

*Note:* For *p* < 0.05, (*) was added after the *p*‐value.

Abbreviation: MoCA, montreal cognitive assessment.

Further analyses indicated that both GFAP and NLRP3 concentrations were significantly correlated with the severity of PWMH and PVS‐CSO (Table 3). After adjusting for these protein levels and covariates, PWMH severity remained significantly associated with MoCA total score and cognitive subdomains including attention, language (*p* < 0.05), and for naming (*p* = 0.069).

**TABLE 3:** Relationship of plasma proteins and small vessel disease imaging biomarkers.

Protein	Imaging biomarkers	r	*p* value	*p* value (FDR)
NLRP3	DWMH	0.087	0.282	0.439
	PWMH	0.292	< 0.001*	0.003*
	Lacunes	−0.058	0.474	0.510
	PVS‐BG	0.156	0.052	0.146
	PVS‐CSO	0.199	0.013*	0.045*
	CMBs	0.141	0.080	0.186
	Summary SVD score	0.128	0.112	0.225
GFAP	DWMH	0.064	0.426	0.497
	PWMH	0.251	0.002*	0.011*
	Lacunes	−0.015	0.848	0.848
	PVS‐BG	0.072	0.372	0.497
	PVS‐CSO	0.208	0.009*	0.042*
	CMBs	0.065	0.422	0.497
	Summary SVD score	0.119	0.139	0.243

*Note:* For *p* < 0.05, (*) was added after the *p*‐value.

Abbreviations: CMBs, cerebral microbleeds; DWMH, deep white matter hyperintensity; PVS‐BG, perivascular space of basal ganglia; PVS‐CSO, perivascular space of centrum semiovale; PWMH, periventricular white matter hyperintensity; SVD, small vessel disease.

**FIGURE 3:**
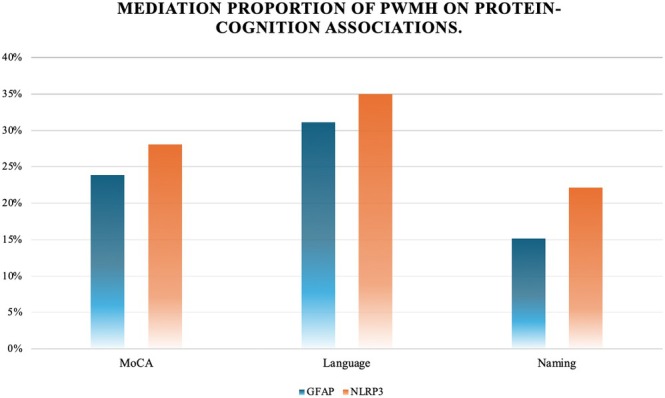
Mediation proportion of PWMH on protein‐cognition associations. The figure shows that PWMH mediated the effects of GFAP and NLRP3 on MoCA, Naming and Language subitems. (MoCA: Montreal Cognitive Assessment, PWMH: Periventricular White Matter Hyperintensity).

Mediation analysis was performed to assess whether PWMH mediated the relationship between inflammatory biomarkers and cognitive outcomes. The results demonstrated that PWMH significantly mediated the relationships between plasma GFAP and NLRP3 levels and both global and domain‐specific cognitive performances (Figure 3).

For GFAP, a significant total effect on overall cognitive function was observed (total effect = −3.16, *p* = 0.008), with an indirect effect (ACME = −0.775, *p* = 0.006) accounting for 23.88% (*p* = 0.010) of the total association. PWMH mediated 28.10% of the NLRP3 effect on the overall MoCA score (ACME = −0.749, *p* = 0.012, for proportion mediated *p* = 0.054).

For cognitive domain‐specific outcomes showing associations with inflammatory biomarkers and imaging biomarkers at a significance threshold of 0.05 < *p* < 0.10, exploratory mediation analyses were subsequently conducted to evaluate the potential indirect effects through PWMH.

For GFAP, significant indirect effects were observed for language (ACME = −0.164, *p* = 0.012; 31.12% mediated, *p* = 0.054) and naming (ACME = −0.098, *p* = 0.040; 15.17% mediated, *p* = 0.042). Similarly, PWMH mediated the effects of NLRP3 on language (ACME = −0.184, *p* = 0.018; 35.02% mediated, *p* = 0.092) and naming performance (ACME = −0.116, *p* = 0.020; 22.15% mediated, *p* = 0.040).

## 4 Discussion

In this study, we found that plasma levels of the inflammatory proteins GFAP and NLRP3 were elevated in patients with RSSI compared to healthy controls. Furthermore, higher concentrations of both proteins were inversely correlated with global and domain‐specific cognitive performance on the MoCA. Notably, PWMH emerged as a potential mediator in the associations between these inflammatory proteins and cognitive impairment. Together, our findings suggest that neuroinflammation‐related astrocytic and inflammasome activation may contribute to cognitive dysfunction by impacting white matter integrity. However, no independent association was found between GFAP or NLRP3 levels and 90‐day functional prognosis.

GFAP, an astrocytic intermediate filament protein, plays a critical role in neuron protection, BBB maintenance, and reactive gliosis [19]. Elevated GFAP expression has been observed in vascular‐related neurological disorders, including both ischemic and hemorrhagic strokes [20]. The TIME trial demonstrated that serum GFAP, in combination with D‐dimer levels, could improve the diagnostic accuracy for large‐vessel occlusion strokes [21]. Genomic studies have revealed that missense mutations in the GFAP gene can alter white matter volume [22]，while clinical and imaging studies in SVD have reported significant correlations between GFAP levels and WMH burden [23]. These associations may be attributed to inflammation‐driven increases in BBB permeability [24] or active demyelination [25], processes that disrupt white matter microstructure and foster cognitive decline. Similarly, the NLRP3 inflammasome, a multiprotein complex, regulates the maturation and release of pro‐inflammatory cytokines such as interleukin‐1β and interleukin‐18 [26]. As a canonical inflammasome element, NLRP3 activation is implicated in ischemic stroke pathology, where its pharmacological inhibition has been shown to reduce infarct volume, dampen inflammatory responses, and improve neurological recovery [27]. Moreover, NLRP3‐mediated inflammatory pathways have been directly linked to SVD pathogenesis in a rat model [12].

Our study extends previous work by demonstrating that peripheral GFAP and NLRP3 are not only elevated after RSSI but are also linked to cognitive deficits. We hypothesized that inflammatory proteins may influence cognitive function by exacerbating white matter lesions. Recent clinical reports have linked elevated NLRP3 expression and WMH burden in patients with SVD, demonstrating their joint association with cognitive impairment and highlighting the potential diagnostic value of both markers in vascular cognitive dysfunction [28]. WMH are thought to arise from chronic ischemia and microvascular dysfunction, processes that involve BBB breakdown, astrocytic reactivity, and subsequent demyelination. Previous histopathological and imaging studies have demonstrated that the progression of PWMH is accompanied by demyelination, gliosis, and axonal loss [29, 30].

Importantly, our mediation analysis indicated that PWMH explains a meaningful fraction of the relationships between inflammatory biomarkers and cognition. It supported the possibility that white matter injury may represent an intermediate pathway linking neuroinflammation and cognitive decline. We hypothesized that inflammatory activation—reflected by elevated GFAP and NLRP3—may initiate or exacerbate BBB dysfunction and myelin damage. Consequently, these could lead to periventricular white matter lesions that impair information‐processing speed and higher‐order cognitive functions. Animal and longitudinal human studies will be required to confirm the temporal ordering and causality of these pathways.

It is noteworthy that the mediating effect was specific to PWMH, with no significant mediation observed for DWMH. This distinction may reflect differing etiologies: DWMH is more classically associated with chronic ischemia and hypoxia, whereas PWMH may be more sensitive to metabolic and inflammatory insults [31]. Supporting this, histopathological studies in SVD patients have revealed marked astrocytic proliferation and increased GFAP expression in periventricular regions, suggesting a greater susceptibility of this area to neuroinflammation [32]. Exploratory domain‐specific mediation—particularly for language and naming—aligns with the anatomical distribution of periventricular association fibers (e.g., arcuate fasciculus, superior longitudinal fasciculus, anterior corona radiata) that subserve linguistic processing and executive attention [33] [34] [35]. By contrast, memory and orientation rely more heavily on medial temporal and limbic structures that are relatively spared by periventricular white matter injury [36].

Regarding functional outcomes, the absence of significant associations between inflammatory biomarkers and short‐term functional measures warrants careful interpretation. Several factors may contribute to this finding. First, patients in the present cohort predominantly experienced minor strokes [37] (median NIHSS score was three), which resulted in generally favorable short‐term functional outcomes and limited score variability. This restricted range may lead to a ceiling effect, thereby reducing the sensitivity of global functional scales to detect subtle biological influences. Moreover, early functional outcomes are largely determined by acute focal neurological deficits, lesion size, and lesion location, whereas cognitive impairment reflects more diffuse and cumulative brain injury. In this context, biomarkers related to neuroinflammation, and small vessel disease may exert a stronger influence on distributed neural networks and white matter integrity than on short‐term motor or functional recovery [38].

To date, numerous therapies have been proposed to mitigate neuroinflammation after stroke. Clinical trials of agents such as Edaravone [39], minocycline, [40] and glucocorticoid [41] have demonstrated their anti‐inflammatory properties. More recently, a GFAP monoclonal antibody (mAb) was shown to modulate the NLRP3/Caspase‐1/GSDMD axis and dampen glial reactivity, thereby inhibiting inflammation and conferring neuroprotection [42]. However, this finding was reported in a glaucoma model, and its potential to ameliorate white matter injury and post‐stroke cognitive decline in humans remains to be evaluated.

Several limitations should be acknowledged. First, plasma GFAP and NLRP3 levels were measured at a single time point within 48 h after admission. This cross‐sectional assessment does not capture temporal dynamics and therefore cannot determine whether sustained elevations or temporal changes in these biomarkers are more strongly related to white matter injury or cognitive outcomes. Even mediation analyses suggested that PWMH may partially mediate the association between inflammatory biomarkers and cognitive performance, and further study is needed to prove the causal pathways. Second, the study sample was recruited from a single geographic region (southwestern China) and was modest in size, which may limit generalizability; external validation in larger, multiethnic cohorts is warranted. Third, while experimental data suggest interplay between GFAP and NLRP3 signaling, our observational data do not resolve whether these biomarkers interact biologically to modulate SVD pathology and cognition, thus, further mechanistic studies are required. WMH burden was assessed using semi‐quantitative Fazekas scores rather than volumetric or automated measures, which may limit sensitivity to subtle structural changes and potentially underestimate mediation effects. Cognitive performance was assessed within one month after stroke onset, while early post‐stroke cognitive testing may be influenced by incomplete neurological recovery, potentially leading to misclassification of long‐term cognitive status.

## 5 Conclusion

In conclusion, this study provides novel evidence that neuroinflammatory proteins GFAP and NLRP3 are associated with cognitive impairment after RSSI, with their effects being mediated by PWMH. These findings underscore the contribution of inflammation and PWMH lesions to cognitive decline in SVD and suggest that interventions targeting astrocytic and inflammasome activation may offer promising avenues to preserve cognitive function in affected individuals.

## Funding

The research was funded by the Joint Funds of the National Natural Science Foundation of China (U24A20690), the National Key R&D Program of China (2023YFC2506603), the National Natural Science Foundation of China (82371322; 82271328, 82301661), the Noncommunicable Chronic Diseases‐National Science and Technology Major Project (2023ZD0504900), and the Science and Technology Projects of Xizang Autonomous Region, China (XZ202501ZY0120), Sichuan Science and Technology Program (No. 2024YFFK0314), Post‐Doctor Research Project, West China Hospital, Sichuan University (No. 2023HXBH007; No. 2024HXBH041).

## Ethics Statement

The study was approved by the Ethics Committee of West China Hospital (2018521).

## Conflicts of Interest

The authors declare no conflicts of interest.

## Data Availability

The data that support the findings of this study are available from the corresponding author upon reasonable request.
